# Providing English and native language quotes in qualitative research: A call to action

**DOI:** 10.1002/nop2.1115

**Published:** 2021-11-01

**Authors:** Ahtisham Younas, Sergi Fàbregues, Angela Durante, Parveen Ali

**Affiliations:** ^1^ Swat College of Nursing Swat Pakistan; ^2^ Memorial University of Newfoundland St. John's NL Canada; ^3^ Department of Psychology and Education Universitat Oberta de Catalunya Barcelona Spain; ^4^ University of Rome "Tor Vergata" Rome Italy; ^5^ University of Sheffield Sheffield UK

**Keywords:** methodology, qualitative research, quotation, research methods, research reporting

## Abstract

**Background:**

When publishing qualitative research in international journals, researchers studying non‐English‐speaking participants provide quotes in English language. This is an issue of increasing concern given the need to be rigorous to represent a diversity of participants within their context, beyond how language (alone) situates them.

**Aim:**

To argue for providing English and native language quotes in qualitative research reports.

**Design:**

Discussion.

**Methods:**

This paper is based on the literature on use of quotes and translation in qualitative research and authors’ experiences of publishing qualitative research.

**Results:**

Provision of native and English language quotes may allow for greater transparency of findings, thereby reflecting that the researchers adequately captured the socially and culturally dependent experiences of participants.

**Conclusions:**

Presentation of findings with eloquent quotes serves as the gateway into the sociocultural experiences of individuals. We argued against the norm of providing translated quotes in qualitative reports and build a case for the provision of native as well as English language quotes to promote cross‐cultural understanding.

## 1 INTRODUCTION

Qualitative research is invaluable to facilitate the exploration and understanding of the experiences of individuals about diverse phenomena across different cultures and contexts (Bhattacharya, 2017; Teherani et al., 2015). Qualitative research is also context‐dependent and offers an emic viewpoint of the social and cultural reality of the participants (Azungah, 2018). Conducting rigorous qualitative research requires researchers to attend to the cultural and social nuances influencing the studied phenomena. Increasing globalization and diversity create a need for more cross‐cultural qualitative research, but language differences between researchers and participants can substantially affect the rigour of this type of research (Nasri et al., 2020). The language of researchers and the participants, and the differences in language arising due to translations and different dialects, can significantly influence qualitative research since they affect conceptualization, data collection, analysis and reporting procedures (Nes et al., 2010).

Researchers conducting cross‐cultural qualitative studies involving non‐English‐speaking participants often collect data in their native language (Santos et al., 2015; Zeb et al., 2021) and then, subsequently, with the help of translators/interpreters or bilingual researchers, they translate these data into English during the stages of data collection, transcription, analysis and reporting (Feldermann & Hiebl, 2019; Helmich et al., 2017; Santos et al., 2015). When writing and publishing qualitative research in international journals, researchers studying non‐English‐speaking participants are required to provide participants’ quotes in English. We argue that this is an issue of increasing concern given the need to be rigorous to represent a diversity of participants within their context, beyond how language (alone) situates them. Translating qualitative data into English is often considered a marker for expediting communication and interaction with a global audience (Abfalter et al., 2020).

Nevertheless, translation of participants quotes can undermine the presentation of context and contextual meanings inherent to participants’ experiences when expressing themselves in a different language. Translation involves interpretation on the part of researchers (Nes et al., 2010). Therefore, because language contains meanings rooted in more than the research context, the precise meaning of research findings may get lost or changed because audiences from different backgrounds may lack information on the specific contexts of the findings (Abfalter et al., 2020; Nasri et al., 2020). Consequently, to promote cultural and language transparency and describe the contextual meanings of participants, there is a greater need to represent the original voices of non‐English language speakers in research reports. Presenting original voices of participants can also enhance the rigour of qualitative research by supplementing knowledge of the researcher's positionality and how it affects the researchers’ interpretation of participants’ experiences. Providing both native and translated quotes has an added potential to enhance the symbolic and conceptual utility of qualitative research. Sandelowski (2004) explains this conceptual utility “as worlds are created with words, and words are the primary currency of qualitative research, to reword something is to remake the world” (p. 1373).

## 2 AIM

To present an argument for providing English and native language quotes in qualitative research reports by outlining the potential benefits of this practice. First, we briefly discuss existing guidance concerning translation in qualitative research. Second, we discuss the importance of providing quotes in qualitative research. Finally, we outline several benefits of providing English and native language quotes in qualitative research and propose recommendations for carrying out this task.

## 3 TRANSLATION IN QUALITATIVE RESEARCH

To date, there has been extensive discussion in the literature about issues concerning the translation of data in qualitative research (Abfalter et al., 2020; Chen & Boore, 2010; Hendrickson et al., 2013; Nasri et al., 2020; Santos et al., 2015). Evidence suggests that translation‐related decisions have a direct impact on the validity of a study's findings and require the researcher to be aware of issues and concerns surrounding translation and interpretations (Chen & Boore, 2010; Squires, 2008, 2009). Many authors have suggested strategies to ensure rigour in translation, and these include: transcribing verbatim in the original language in which data were collected, using two bilingual researchers, moving back and forth between raw and translated data to ensure consistency, choosing the appropriate time to do the translation, being mindful of the conceptual equivalence when translating, enabling sociocultural matching and ensuring that researchers are linguistically competent and culturally sensitive (Chen & Boore, 2010; Hendrickson et al., 2013; Nasri et al., 2020; Santos et al., 2015). To enhance the transparency of translation decisions and improve the scientific rigour of translated qualitative studies, Abfalter et al. (2020) proposed a framework making use of the following generic queries:

### 3.1 WHY? The reason for translating

Researchers should clarify the reasons for translating the quotes. The common reasons are linguistic comfort, value for the academic community and academic career. Linguistic comfort refers to the idea that researchers and participants may feel at ease when interpreting and sharing their data in the native language. However, the researchers then need to translate the quotes, so the global academic community can benefit from the research. Translation of quotes may enable researchers to expand the epistemological spectrum by presenting distinct sociocultural phenomena and their variations concerning the characteristics of individuals in those contexts. This notion refers to the value for the academic community. Finally, researchers may translate their qualitative research and quotes into English because international publications are required for academic promotion in many countries (Abfalter et al., 2020).

### 3.2 WHEN? The time for translating

Researchers should clearly note the timing for translation during the research process. Early‐stage translation of data can ensure homogeneity of concepts and language and may save the cost of translation. However, translation at a later stage can produce higher‐quality findings because researchers can discover new insights while analysing the data in their native language. Nevertheless, later stage translation can be costly and time consuming (Abfalter et al., 2020).

### 3.3 WHAT? The data or content for translating

It is important to clarify what content is to be translated because limited complexity in translation can result in greater loss of meaning and context of the original data. Some researchers may only translate the verbal data, while others may be interested in verbal data and emotions, exclamatory remarks, humour, metaphors, traditional and cultural expressions, and other linguistic characteristics (Abfalter et al., 2020).

### 3.4 WHO? The person(s) translating

The person responsible for translating the data has the authority and responsibility to make pertinent decisions about the translation process and the content. The research teams should clearly note who (eg research team, data analyst, research assistant, interpreter or any other person outside the research team) will be involved in translation (Abfalter et al., 2020).

### 3.5 HOW? The mode of translating

Researchers should state the techniques used for translation because the diversity and heterogeneity of languages of the participants can affect the translation process. Abfalter et al. (2020) discussed two techniques. First, a contextualized hermeneutic approach ensures the accurate translation of the meaning and experiences of participants. Second, a technicist approach follows strict rules and methods to translate the data for enhancing its accuracy, validity, reliability and quality.

### 3.6 WHERE? The location for translating

The location for translation could be (i) “within the socio‐geographical environment of the source language, (ii) within the socio‐geographical environment of the target language and (iii) outside the socio‐geographical environment of both source and target language” (Abfalter et al. (2020), p. 12). Translation within the source language or native language in the specific subculture can preserve contextual information and meaning of participants’ experiences. Translation within the target language, that is, English, may affect the researcher's positionality, social identity and the contextual meaning of the data (Abfalter et al., 2020).

### 3.7 BY WHAT MEANS? The means and tools for translating

Researchers should also explicitly describe the tools and methods used translating data from native to target language. The tools may include online software, glossaries, internet searches and IT tools (Abfalter et al., 2020).

This framework is useful for qualitative researchers engaged in cross‐cultural research for appropriate translation and integration of language to capture participants’ experiences. We recommended utilizing this framework during the research process and tailoring the queries to the context of each study.

## 4 IMPORTANCE OF PROVIDING QUOTES IN QUALITATIVE RESEARCH

Researchers must work consciously to ensure the rigour of the translation of qualitative data and the integrity of their decision‐making when presenting their findings. One quality criterion essential to the reporting of qualitative research is the inclusion of participants’ quotes in the findings section of the study (Malterud, 2001; Walsh & Downe, 2006). Quotes are presented as “evidence, explanation, illustration, impression, representation and/or to enhance the readability of qualitative research” (Eldh et al., 2020, p. 5). Using quotes allows researchers to provide strong evidence that the reported findings are credible and accurate representations of the participants’ views and experiences (Eldh et al., 2020; Fitzpatrick & Boulton, 1996). Embedding participants quotes within paragraphs of theme description adds additional richness to the study findings (Eldh et al., 2020), allows the reader to see the issue from the participants’ perspective in their own words and helps strengthen the reporting of participants’ experiences when interpreting results, particularly when authors are dealing with a community, they are not a part of.

## 5 CHALLENGES WITH THE PRESENTATION OF QUOTES IN ENGLISH AND THE NEED FOR CHANGING THIS PRACTICE

As previously discussed, authors have mainly argued for ensuring transparency and rigour during the translation of qualitative data (Chen & Boore, 2010; Nasri et al., 2020; Santos et al., 2015). Such arguments are presented with the underlying assumption that English readers can understand and attest to the accuracy and rigour of data analysis and reporting (Al‐Amer et al., 2015). However, attention needs to be paid when information is translated from one language to another and the nuances of the message conveyed via words—not to mention accent, dialect and ways of speaking—are lost. This practice can supress the culturally flavoured meanings of individuals’ experiences and views (Helmich et al., 2017).

To adequately and rigorously present results, researchers working with non‐English participants often provide participants’ quotes in English when writing and publishing qualitative research for international journals. The quotes are provided as if the participants shared their experiences and views in fluent English, assuming that readers will be able to adequately capture the underlying meanings (Al‐Amer et al., 2015; Helmich et al., 2017). Even when the original data are in English, researchers often produce a corrected version of the participants’ quotes by tidying up any mispronunciations and removing common colloquial expressions such as “humms” and “aahs” (King et al., 2019). While this practice can help make the quotes more comprehensible and readable, it can also affect transcription accuracy and reduce the authenticity of the reported data. These two problems can be still more significant when quotes are translated since researchers might then need to do further editing to make the text more comprehensible. Esposito (2001) argued that some types of translations might lead researchers to lose the “emic quality” of the original dialogue between the participants and the researchers. Translating quotes might make it difficult to preserve the original meaning of the qualitative data when reporting the study findings.

Sandelowski (1994) proposed two approaches to the reporting of the participants’ quotes: the “preservationist” approach and the “standardized” approach. While in the preservationist approach, researchers try to preserve every element of the participants’ verbal quote, in the standardized approach, researchers clean up every textual element that might be distracting to the readers. An example of the latter approach is provided by Morse (1996), who argues that researchers should edit the participants’ quotes because, in her own words, unedited quotes “will distract the reader, and the message that the researcher is trying to convey will be obscured by the irrelevant material.” In this article, we argue that the time has come that, in cross‐cultural qualitative reports, a preservationist approach is prioritized over a standardized approach. Nevertheless, it is also important that the choice of the preservationist approach aligns with the study purpose and researchers’ paradigmatic and methodological stance. The preservationist approach can be adhered to by encouraging researchers to provide participants’ quotes in both English as well as the original language of the data collection. While providing both native and English language quotes, the researchers should also discuss the reasoning for choosing and presenting quotes. It is also important that journal editors encourage and support the presentation of English language quotes alongside translated quotes. The inclusion of bilingual quotes can help highlight the voices of participants living in non‐English‐speaking countries. Since those participants might be less prevalent than English native speakers in studies published internationally, the inclusion of bilingual quotes could be understood as a way of democratizing research (ie reducing imbalances of power within the research process).

## 6 POTENTIAL BENEFITS OF PROVIDING ENGLISH AND NATIVE LANGUAGE QUOTES’

### 6.1 Contribution to rigour in data analysis

Qualitative data analysis is a daunting task that requires researchers to practice creativity, innovation, intuition and abductive reasoning. In cross‐cultural qualitative research, it is essential that the participants’ experiences are adequately interpreted within their contexts (Pelzang & Hutchinson, 2017; Saldaña, 2021) and, as a result, participants’ experiences are converted into meaningful themes to inform practice. The presentation of qualitative findings needs to be done in descriptive and interpretive ways so that the meanings of the studied phenomena are illuminated in the light of the social and cultural contexts of the phenomena (Munhall, 2012).

Describing the themes in qualitative research, the provision of direct quotations enables researchers to grasp the data in an authentic way, however, assuming that the readers’ evaluation of the rigour and plausibility of the study is largely contingent on the presented data (Feldermann & Hiebl, 2019). Therefore, the provision of both native and English language quotes in the qualitative report may allow multilingual readers to assess the consistency and adequacy of the translation and the extent to which the quotes accurately represent the themes. Consequently, readers may develop greater confidence in the study findings. For example, a reader who is proficient in both Italian and English languages may find a qualitative report with Italian and English language quotes more relevant than a similar study that only provided quotes in English. This could be due to various reasons: (i) readers can better relate to the former qualitative report than the later, (ii) readers can better evaluate the consistency between the actual experience of participants and the presented account of the participants’ experience, and (iii) readers can better relate to and contextualize the views and experiences of the participants in the broader social and cultural context.

### 6.2 Greater transparency in research reporting

Transparency in qualitative research entails comprehensive reporting of study methods, researchers’ assumptions and biases, cultural context and study findings (Tuval‐Mashiach, 2017). In reporting study findings, participants’ quotes are crucial to support the themes, their interpretation and explanation, and to convey the explicit meaning of the generated themes (Yin, 2011). Put simply, the presentation of the participants’ quotes gives voice to the generated themes (Sutton & Austin, 2015) and provides vividness to the text (Eldh et al., 2020). Participants’ words and phrases demonstrate the depth of their views and the intensity and underlying emotions of their feelings and experiences. Although researchers may use emotional labels such as anger, distrust, disgust and surprise to adequately describe participants’ experiences, quotes are better illustrative of the participants’ true feelings (Corden & Sainsbury, 2006). Therefore, if the participants shared their experiences in their native language, it seems reasonable that the quotes are presented as such.

Relevant and meaningful participants’ quotes can potentially enhance the representation of their experiences, the evocation of readers’ emotions and the process of generating responses (Bradshaw et al., 2017; Sandelowski, 1994). Sometimes, participants’ actual words can better impact on readers compared to researchers’ descriptions of those experiences (Corden & Sainsbury, 2006). No matter how accurate the translation can be, there is always a possibility of losing the true essence and cultural and social favour in a translated quote. An inaccurate and inadequate translation may take away the essential flavour of the quote that makes it appealing and interesting to the readers. Therefore, if both native and English language quotes are provided, it adds transparency to language reporting for the readers. As discussed previously, multilingual and native readers can assess the adequacy of translation and the relevance of the quotes to the themes.

### 6.3 Ensuring adequate capture of participants’ views

Quotes presented in qualitative research need to illustrate the essence of the participants’ experiences and “bring the text to life—or bring life to the text” (Eldh et al., 2020, p. 4). Relevant and relatively succinct quotes demonstrate points that researchers make about the data and provide insights into the global and contextual patterns in the data (Lingard, 2019). When quotes are translated, errors of omission and translation and differences in translated texts can substantially affect the interpretation of the data, the generation of meanings and the final representation of the participants’ experiences (Wong & Poon, 2010).

The above‐listed translation errors could make researchers fail to adequately capture participants’ views in studies conducted in settings with ingrained cultural values and norms related to race, gender, stigma and marginalization (Nasri et al., 2020). In those types of studies, readers may expect greater awareness of the inherent cultural norms and values, and such awareness is often reflected in the quotations presented in the published qualitative findings. Publishing quotes in the original language may offer the opportunity for the readers to gather insights into the essence of the participants’ experiences. It might also allow researchers to retain an insider's perspective and ensure that the participants’ views are accurately captured and represented in their own words (Manning, 1997; Patton, 2002). Finally, reporting quotes bilingually might give readers a chance to immerse themselves in the situations that the quotes relate to and develop accurate insights about the participants’ social and cultural contexts.

### 6.4 Promoting cross‐cultural understanding

One of the aims of cross‐cultural research is to offer readers an understanding of the complexities of culturally and socially dependent phenomena and recognize the differences in phenomena across contexts (Karasz & Singelis, 2009). Greater consideration must be given to research methods in cross‐cultural research conducted in collectivistic cultural settings entailing greater sense of participants’ socio‐political dynamics (Pelzang & Hutchinson, 2017). Language differences are integral to understanding collectivist diversity across contexts and cultures. Language is not just words, but also a symbolic system of communication shaped by the customs, beliefs, identities, and world views of a given social group (Angel, 2013).

Each language has its own metaphors, and analogies and embodies figures of speech that are filtered through the social consciousness of the speakers. For example, the word “solicitor” means salesperson in the USA, but lawyer in the UK (Cambridge Dictionary, 2020). If an English word has different meanings across English‐speaking countries, it also has greater variations in meaning in other languages. The word “acha” could have several meanings within Urdu and Hindi languages based on the connotation and context. The sentence “Ye acha hai” (acha used in a positive connotation) means “this is good” in English, but “Acha, ab tum mujhe samjhogy kai ye kam kese hota hai” means “OK, are you going to teach me how to do this now/ do I have to learn this from you now?” (Acha used in an aggressive connotation). Or the same sentence “Ye acha hai?” can be used in a sarcastic way (sarcastically saying this is great!). Similarly, Nes et al. (2010) exemplified that the “*gezellig*” is used in Dutch language by late‐life couples, while expressing the feeling they had when doing things together. The meaning expressed with this Dutch word entails “experiencing togetherness in doing everyday activities together, often at specific times of the day and in the own home. Translating the word gezellig, only as ‘cosy’ would reduce the meaning” (Nes et al., 2010, p. 315). Many of these differences can be found in other languages, as illustrated in Table 1. Therefore, reporting participants’ quotes in English and their native language can help address the problem of words’ multiplicity of meanings across languages while offering readers the opportunity to develop cross‐cultural understanding of participants’ accounts. Readers who are only proficient in English may develop an interest in learning more about the native language of the participants. When the native language is made available along with the translated text, readers have a greater opportunity to appreciate the described cultural phenomenon and apprehend the richness embedded in the study findings.

**TABLE 1:** Examples of lost meaning during translation

Language	Word/phrase/expression	Lost meaning
Urdu	The phrase “Larkiyan hamari izzat hain” is often used by people with conservative religious, traditional cultural and patriarchal beliefs to refer to the idea that women in their families are their honour and therefore they must avoid any culturally unacceptable practices that may affect the social reputation of their families.	When this phrase is literally translated into English, it means “Girls are our honour”. This translation results in complete loss of the essence and context of the phrase. Girls are our honour can be interpreted in various different ways if the context is lost. For example, some readers may interpret that this phrase acknowledges the status or role of women in the family, while others may interpret that women are most honourable members of the families.
Spanish	The expression “está más seco que la mojama” is used in Spain to describe an extremely thin person, including someone unhealthy with a low body mass index.	“Mojama” is a Mediterranean delicatessen consisting of dried filleted salt‐cured tuna, typical from the provinces of Cadiz and Huelva in Spain. Since the word “seco” (dry) is used in Spain to describe a thin person, the expression means that someone is even dryer than the “mojama”. Given the contextual nature of this expression, it is highly likely that its meaning will not be fully captured by readers from non‐Mediterranean countries or even from other countries than Spain.
Italian	The phrase “rubare con gli occhi” is used to express the concept to learn by watching a professional doing his/her own job. Informally, it can be referred also to learning by somebody in the family for learning some tricks like in cooking (by mothers for examples).	In English, it can be translated as “work shadowing” which means the activity of spending time with someone who is doing a particular job so that you can learn how to do it. The example reported is taken by an informal/family caregiver. It is referred on how to act after hospital discharge, “stolen” to nurses during the hospitalization. Despite that the concept looks similar, there is a sensible cultural difference which is given by the informal context in which the “learned”, without a proper education, will be performed with the consequence of being inappropriate or at least dangerous.

## 7 CONCLUSIONS AND RECOMMENDATIONS

Presenting participants’ quotes in qualitative research reports is critical for enhancing the rigor, interpretation and explanation of research findings. In cross‐cultural qualitative research, the presentation of comprehensive findings with eloquent quotes essentially serves as the gateway into the culturally and socially dependent experiences of individuals. In this article, we argue against the common practice of providing translated quotes in qualitative research reports. We build a case that the provision of native and English language quotes may allow for greater transparency, rigor and cross‐cultural understanding of research findings.

We are also conscious of the fact that sometimes journals do not have adequate space to publish qualitative studies with a long list of quotes. Therefore, we offer three recommendations to overcome this limitation. First, the translated and original quotes could be presented in a supplementary online file, only if the journal restricts the word count. If the journal space permits, the authors should provide both English and original language quotes under each theme or sub‐theme in the research report. Second, as Bazeley (2013) suggested, researchers working in a second language can “use occasional phrases (or brief quotes) from the original language in the final report as needed, accompanied by a parenthesized translation or an explanation where you can't directly translate” (p. 77). Such publishing practices are already common in the field sociolinguistics. For example, the *Journal of Sociolinguistics and Journal of Applied Linguistics* (published by Wiley) requires authors to provide English and non‐English quotes and encourages adding a second abstract in the authors’ native language to avoid losing the context of findings. Therefore, it seems reasonable to provide both English and native quotes when reporting qualitative studies in the fields of nursing and the health sciences. Finally, the authors of cross‐cultural qualitative studies should explicitly discuss any potential challenges and issues encountered during the translation and analysis of native language quotes in their manuscripts. It is important that readers are aware of the issues that may have affected the transparent reporting of qualitative quotes and their underlying contextual meanings.

## CONFLICT OF INTEREST

Dr. Parveen Ali is an Associate Editor for Nursing Open.

## Data Availability

Data sharing is not applicable to this article as no datasets were generated or analysed during the current study.
